# Thirteen metagenome-assembled genomes of *Paraglaciecola chathamensis* associated with the farmed red seaweeds *Porphyra dioica* and *Porphyra umbilicalis*

**DOI:** 10.1128/mra.01496-25

**Published:** 2026-03-10

**Authors:** André C. Pereira, Francisco Cortez, Guilherme Chaves, Enrico Nanetti, Ricardo B. Leite, Madalena Caria Mendes, Inês Oliveira, Helena Abreu, Margarida Martins, Tina Keller-Costa, Rodrigo Costa

**Affiliations:** 1 Institute for Bioengineering and Biosciences (iBB) and Institute for Health and Bioeconomy (i4HB), Instituto Superior Técnico, Universidade de Lisboa, Lisbon, Portugal; 2 Department of Bioengineering, Instituto Superior Técnico, Universidade de Lisboa 37809, Lisbon, Portugal; 3 Unit of Microbiome Science and Biotechnology, Department of Pharmacy and Biotechnology, University of Bologna 9296, Bologna, Italy; 4 Instituto Gulbenkian de Ciência (IGC) 705996 , Oeiras, Portugal; 5 ALGAplus, Production and Trading of Seaweed and Derived Products S.A, Ílhavo, Portugal; 6 GreenCoLab, Associação Oceano Verde, Universidade do Algarve 70985, Faro, Portugal; 7 Centre for Environmental and Marine Studies (CESAM), Department of Chemistry, University of Aveiro 56062, Aveiro, Portugal; 8 Bantry Marine Research Station, Gearhies, Cork, Ireland; California State University San Marcos, San Marcos, California, USA

**Keywords:** *Paraglaciecola*, Atlantic Nori, marine symbioses, host-microbe interactions, algal microbiomes, bacterial genomics, *Porphyra*, aquaculture

## Abstract

We report 13 metagenome-assembled genomes (MAGs) of *Paraglaciecola chathamensis* (*Gammaproteobacteria*) retrieved from farmed Atlantic Nori (*Porphyra* spp.) across several developmental stages. MAGs encode proteins involved in host-microbe interactions, nutrient acquisition, nitrogen and cofactor metabolism, stress resilience, and genome plasticity, illuminating the possible roles of *Paraglaciecola* in the *Porphyra* holobiont.

## ANNOUNCEMENT

Atlantic *Porphyra* spp. (Atlantic Nori) are red macroalgae of economic importance, particularly as a food source (1, 2). The microbiota of macroalgae plays a key role in holobiont growth and development, with the bacterial genus *Paraglaciecola* reported as part of the *Porphyra* microbiome (3, 4). The role of this taxon in algal-microbiome associations is, however, poorly understood (5–8). We report thirteen metagenome-assembled genomes (MAGs) of *Paraglaciecola chathamensis* retrieved from *Porphyra* spp. and culturing seawater from a land-based integrated multi-trophic aquaculture (IMTA) system, increasing by more than 2-fold the number of publicly available genomes in DDBJ/ENA/GenBank for this species.

The MAGs originate from replicate, individual algal tissue (*Porphyra dioica* and *Porphyra umbilicalis*, 2 g fresh weight) and culturing seawater (1 L) samples collected aseptically at ALGAplus IMTA facilities (Ílhavo, Portugal, 40°36′46.3″N, 8°40′*2*6.5″W) on November 9, 2023 (*P. dioica*) and March 21, 2024 (*P. umbilicalis*) (9), encompassing four algal biomass production stages: conchocelis (S1), conchosporangia (S2), young blades (S3), and mature blades (S4). While S1–S3 algae are cultivated in indoor photobioreactors with autoclaved IMTA seawater, S4 algae are cultivated outdoors with IMTA seawater (for details, see Cortez *et al*. [9]). Seawater samples were filtered through sterile 0.22-μm mixed cellulose membrane filters (Merk, Germany). Total community DNA was extracted from individual algal samples (0.5 g wet biomass) and 0.22 μm filters using the DNeasy PowerSoil kit (QIAGEN, Germany). DNA libraries were prepared using the MGIEasy Fast FS DNA Library Prep Set (MGI, China) and subjected to paired-end metagenome sequencing (MGI G400 with a large flow cell and a PE150 cartridge). The raw sequence reads were quality checked with FastQC (v.0.12.1) (10), filtered and trimmed with fastp (v.1.0.1) (11), and mapped against the *P. umbilicalis* genome (GCA_002049455.2) with BWA-MEM2 (v.2.3) (12). Non-mapped reads were taxonomically classified with Kraken2 (v.2.1.3) (13) using the PlusPF database (v.2024-01-12). Reads that were mapped to the *P. umbilicalis* genome or classified as Metazoa were removed. Metagenome assembly was performed, per sample, for the remaining reads using metaSPAdes (SPAdes v.4.2) (14). Metagenome-assembled genomes (MAGs) were then binned from the obtained assemblies with MetaBAT2 (v.2.17) (15), quality checked with CheckM (v.1.2.4) (16), and taxonomically classified with GTDB-Tk (v.2.4.1, R220 database) (17). FastANI (v.1.3) (18) was used to compute whole-genome average nucleotide identity (ANI). Here, we report 13 MAGs identified as *Paraglaciecola chathamensis* showing high completeness and low contamination estimates (Table 1). Contigs were annotated using the NCBI Prokaryotic Genome Annotation Pipeline (PGAP v.6.10) (19). Clusters of Orthologous Groups of proteins (COGs) and Carbohydrate-Active Enzyme (CAZyme) annotations were further performed using Melange (https://github.com/sandragodinhosilva/melange). Default parameters were used for all software.

**TABLE 1:** General features of metagenome-assembled genomes (MAGs) of *P. chathamensis* obtained from *Porphyra* spp. and surrounding seawater

Metagenome-assembled genome^*a*^	Completeness (%)^*b*^	Contamination (%)^*b*^	Genome size (bp)	Genome coverage (X)	No. of contigs	Contig N50 (bp)	GC content (%)	ANI (%)^*c*^	AF ^*c*^	No. of coding sequences^*d*^	No. of genes^*d*^	No. of RNAs^*d*^	No. of rRNAs^*d*^	No. of tRNAs^*d*^	No. of ncRNAs^*d*^	No. of COGs^*e*^	No. of CAZymes^*e*^	GenBank accession no.	Biosample accession no.	SRA accession no. (original metagenome)	No. of reads in original metagenome
Pdi_S3a_mag4	73.53	4.08	4,219,731	7.53	686	8,691	44.3	98.73	0.889	4,071	4,105	34	0	31	3	2,879	58	JBSVKR000000000	SAMN52734530	SRR35789804	17,346,767
Pdi_S3b_mag9	88.44	6.00	5,149,040	31.71	348	25,576	44.3	98.83	0.841	4,522	4,554	32	0	28	4	3,402	82	JBSVKS000000000	SAMN52734531	SRR35789803	33,378,705
Pdi_S3c_mag7	84.97	3.21	4,667,620	25.46	273	27,746	44.4	98.83	0.930	4,091	4,117	26	0	22	4	3,190	78	JBSVKT000000000	SAMN52734532	SRR35789854	26,067,503
Pu_S2a_mag9	97.98	1.11	4,009,856	11.61	501	13,525	44.3	98.70	0.943	3,807	3,851	44	1	39	4	2,816	60	JBSVKU000000000	SAMN52734533	SRR35789848	37,329,787
Pu_S2b_mag3	76.66	2.07	4,059,528	12.58	477	13,992	44.3	98.70	0.941	3,809	3,840	31	0	28	3	2,854	59	JBSVKV000000000	SAMN52734534	SRR35789847	41,095,954
Pu_S2c_mag6	96.03	0.00	3,978,846	26.31	457	13,950	44.4	98.75	0.949	3,660	3,695	35	0	32	3	2,773	61	JBSVKW000000000	SAMN52734535	SRR35789846	42,047,204
Pu_S2d_mag1	96.35	0.00	3,922,693	21.55	457	13,956	44.3	98.78	0.949	3,643	3,676	33	0	30	3	2,739	64	JBSVKX000000000	SAMN52734536	SRR35789845	29,206,649
Pu_S3a_mag7	92.43	0.63	3,598,380	11.15	519	10,262	44.4	98.69	0.967	3,453	3,491	38	0	35	3	2,557	52	JBSVKY000000000	SAMN52734537	SRR35789843	33,528,794
Pu_S3b_mag24	93.65	0.90	3,775,791	11.12	451	12,619	44.4	98.71	0.963	3,564	3,601	37	0	34	3	2,683	51	JBSVKZ000000000	SAMN52734538	SRR35789842	36,951,890
Pu_S4d_mag3	94.78	0.56	3,821,571	10.34	502	11,869	44.4	98.65	0.958	3,618	3,663	45	0	42	3	2,699	55	JBSVLA000000000	SAMN52734539	SRR35789814	33,600,272
SWPdi1a_mag7	81.79	1.37	4,236,573	38.53	367	20,020	44.3	98.70	0.935	3,874	3,911	37	0	33	4	2,988	61	JBSVLB000000000	SAMN52734540	SRR35789839	47,916,948
SWPdi1b_mag3	90.47	2.61	3,754,556	12.52	555	9,671	44.3	98.75	0.910	3,579	3,599	20	0	18	2	2,606	56	JBSVLC000000000	SAMN52734541	SRR35789838	35,119,860
SWPdi3b_mag9	94.71	0.83	4,001,220	14.87	537	11,474	44.4	98.84	0.924	3,729	3,757	28	0	24	4	2,765	62	JBSVLD000000000	SAMN52734542	SRR35789834	41,314,761

^*a*^ Individual MAGs are labeled according to their biotope of origin (Pu, *Porphyra umbilicalis*; Pdi, *Porphyra dioica*; SW, culturing seawater), developmental stages of *Porphyra *spp., from S1 to S4, and the individual replicate samples they originate from (a, b, c, or d). For instance, “Pdi_S3a” describes a MAG retrieved from a *Porphyra dioica* sample at developmental stage S3, biological replicate “a”; “SWPdi1a” describes a MAG obtained from *P. dioica* culturing water at developmental stage S1, seawater replicate “a”. S1, Conchocelis stage produced in photobioreactors indoor; S2, Conchosporangia stage produced in photobioreactors indoor; S3, Young blade stage produced in photobioreactors indoor; and S4, Mature blade stage produced in photobioreactors outdoor.

^*b*^ Completeness and contamination estimates according to the NCBI Prokaryotic Genome Annotation Pipeline (PGAP v.6.10).

^*c*^ Average nucleotide identity (ANI) and alignment fraction (AF) were obtained with CheckM against the genome sequence of type strain *P. chathamensis* (accession number GCF_000314955.1).

^*d*^ Annotation of basic genome features was performed using the NCBI Prokaryotic Genome Annotation Pipeline (PGAP v. 6.10): https://www.ncbi.nlm.nih.gov/genome/annotation_prok/.

^*e*^ The Melange pipeline (https://github.com/sandragodinhosilva/melange) was used to perform Clusters of Orthologous Groups of proteins (COG) and Carbohydrate-Active Enzymes (CAZymes)-based annotations. The corresponding data are available on Zenodo under DOI: https://doi.org/10.5281/zenodo.17911112.

**Fig 1:**
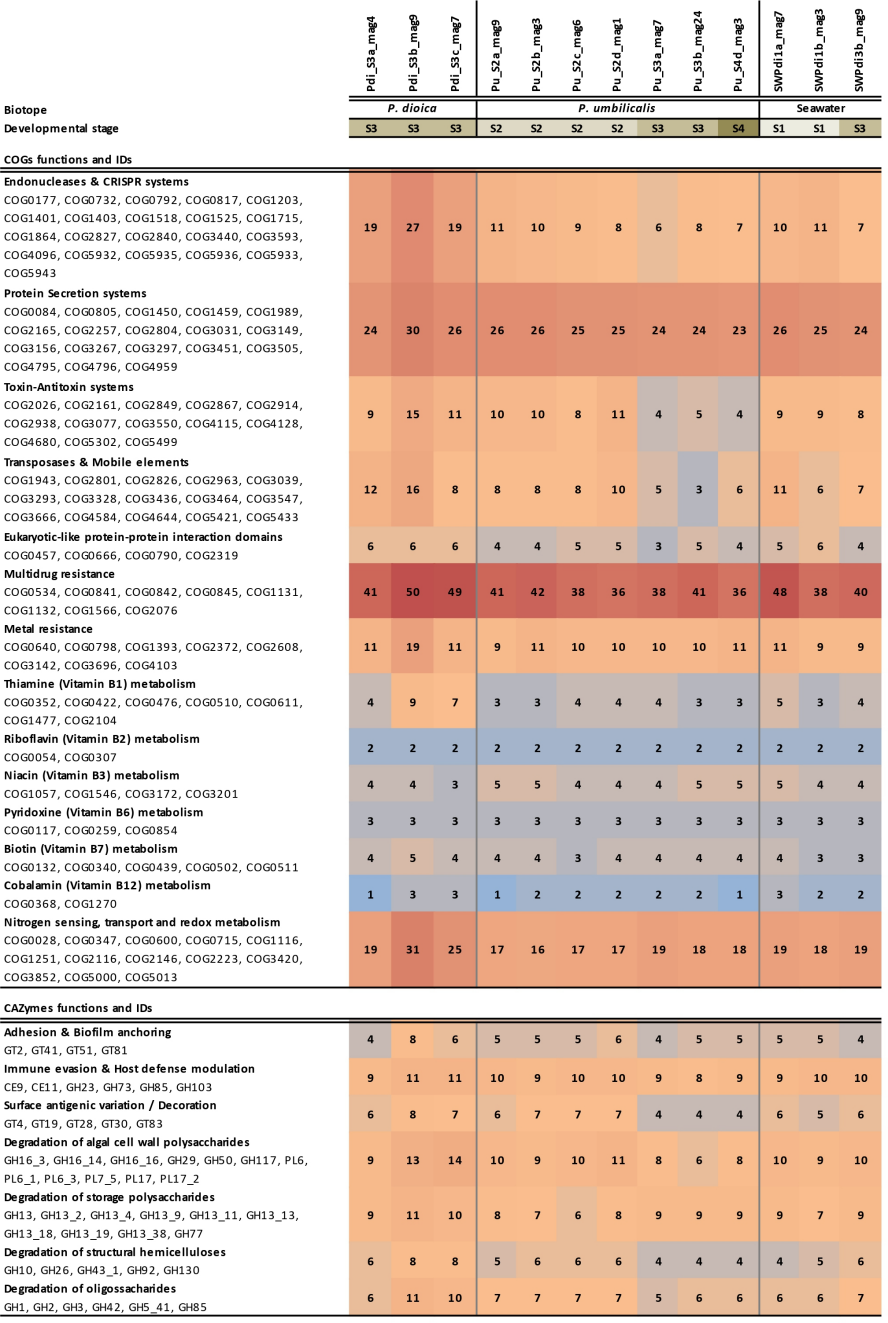
Selected Clusters of Orthologous Groups of proteins (COGs) and Carbohydrate-Active Enzymes (CAZymes) functions characteristic of the *P. chathamensis* genomes described in this study. Values for each entry represent the sum of the respective COGs (top) and CAZymes (bottom) functions per genome. Individual MAGs are labeled according to their biotope of origin (Pu, *Porphyra umbilicalis*; Pdi, *Porphyra dioica*; SW, culturing seawater), developmental stages of *Porphyra* spp., from S1 to S4, and the individual replicate samples they originate from (a, b, c, or d). For instance, “Pdi_S3a” describes a MAG retrieved from a *P. dioica* sample at developmental stage S3, biological replicate “a”; “SWPdi1a” describes a MAG obtained from *P. dioica* culturing water at developmental stage S1, seawater replicate “a”. S1, stock mother cultures of conchocelis growing vegetatively; S2, conchocelis cultures where the differentiation to the conchosporangia stage takes place; S3, vegetatively growing young blades; and S4, vegetatively growing mature blades.

The 13 *P. chathamensis* MAGs shared ≥98.7% ANI among themselves and with the genome of the type strain *P. chathamensis* S18K6 (GCF_000314955.1). Several genes are related to host-microbe interactions (e.g., eukaryotic-like proteins), remodeling of glycoproteins, and persistent colonization of surfaces (e.g., extracellular polysaccharide production) (Fig. 1). A diverse set of glycosyl hydrolases presumably enables the degradation of *Porphyra* polysaccharides (floridean starch, porphyran, agar, and carrageenan) by *P. chathamensis*, ensuring efficient nutrient acquisition. Nitrogen assimilation and redox metabolism, together with vitamin biosynthesis pathways (biotin, thiamine, riboflavin, cobalamin, and pyridoxine), may contribute to holobiont nutrition. Mechanisms for multidrug and metal resistance and genome plasticity likely promote resilience and ecological versatility of *P. chathamensis* populations in dynamic host-microbe scenarios. MAGs from *P. umbilicalis* blades (S3/S4) displayed lower counts of toxin/anti-toxin systems, transposases, and mobile elements than those recovered from *P. umbilicalis* conchosporangia (S2).

## Data Availability

The metagenome-assembled genomes, as well as the raw metagenome data, have been deposited at DDBJ/ENA/GenBank under the BioProject PRJNA1345216 and SRA accession numbers SRR35789803, SRR35789804, SRR35789814, SRR35789834, SRR35789838, SRR35789839, SRR35789842, SRR35789843, SRR35789845-SRR35789848, SRR35789854. The BioSample accession numbers of the MAGs are SAMN52734530- SAMN52734542, and their GenBank accession numbers are JBSVKR000000000-JBSVLD000000000. The MAGs were obtained from the *Porphyra* and seawater metagenome BioSamples SAMN50730953-SAMN50730955, SAMN50730961-SAMN50730966, SAMN50730969, SAMN50730970, SAMN50730974, SAMN52664634. CAZyme and COG annotations are available on Zenodo under DOI: https://zenodo.org/records/17911112.
